# The Institutional Library

**Published:** 1920-12-04

**Authors:** 


### THE INSTITUTIONAL LIBRARY.

**Manual of Medicine.** By A. S. Woodwark, C.M.G., M.D., F.R.C.P. Second Edition. (Hy. Frowde and Hodder & Stoughton. 1920. Pp. 487. Price 16s. net.)

Crammed with the cardinal facts of medicine, this is a very useful book alike for students and practitioners. To the former, faced by an increasingly burdened curriculum, such works would appear to be an established necessity. The discerning student, however, will wisely refuse to treat them as other than adjuncts to the elaborate "systems." To those who have soaked their minds in the wisdom of the great masters of medicine, the book before us, read carefully through during the month preceding the examination in medicine, should prove a real means of grace at the fateful hour. The mere crammer of facts may survive the dogmatic trial of the examination hall, but in after years, when dealing with an increasingly discerning public, he will have cause to bemoan his partial knowledge and limited outlook.

The practitioner who, in moments of doubt and difficulty, requires advice clear and terse, may turn to this volume confident of finding his need met. The book is thoroughly up to date, containing a sufficient digest of the knowledge gained by the author during the late war. Men have returned from foreign service bearing in their bodies the marks of pathological conditions with which the practitioner is in many cases unfamiliar. These he has to deal with, and he may glean knowledge and guidance from these pages.

Written by a physician of established position and great experience, and embodying "authoritative information whilst omitting any but salient features," we can, after carefully perusing the book, recommend it as a trustworthy statement of the essential facts of medicine. As a book it is well arranged, with an adequate index.

**Surgical Nursing.** By Russell Howard, C.B.E., M.S., F.R.C.S. Fourth Edition. Pp. 320. (London: Edward Arnold. 1920. Price 7s. 6d.)

Mr. Russell Howard pays a graceful tribute to the matron and sisters of the London Hospital by dedicating to them the fourth edition of his text-book on "Surgical Nursing." The work contains valuable information from both the practical and theoretical standpoint, the chapters are neatly divided into paragraphs, and the definitions of terms are clear and concise.

The author covers a wide field in "Surgical Nursing," and in places the texture of his work is uneven. He, like many other surgeons, seems little impressed with the merits of the Fowler position, though there are cases for which it has undoubted value. In his treatment of fractures Mr. Howard ignores the lessons taught us during nearly five years of war. The chapter devoted to ophthalmic nursing is ineffectual; many of the statements made are misleading, and the teaching in several instances is in contradistinction to accepted methods. There are few surgeons to-day who would be content with the scalding of dressing bowls; if sterilisation by boiling is not possible, they should be immersed for several hours before they will be required in a solution of carbolic acid. The directions for nasal feeding, the chapters descriptive of bandaging and the surgical nursing of infants attain a high standard of practical value; whilst in the chapter on massage the author successfully approaches the subject from the standpoint of the trained nurse who is not a certificated masseuse.

There is an excellent appendix, and the work is admirably illustrated with photographs and diagrams.

**Organisation of Public Health Nursing.** By Annie M. Brainard. (New York: The Macmillan Co. 1919. Price 7s. 6d. net.)

The authoress well remarks in her foreword that "public-health nursing is a profession still in process of evolution." It is for this reason that we trust a cordial welcome will be extended in this country to a record of organisation far beyond anything hitherto attempted in the British Isles. We may smile at the earnest recommendation that public-health nursing should be kept out of politics, and rejoice that here at any rate the office of the public-health nurse is not a political plum to be used as a reward for services rendered in an electioneering campaign, but we have much to learn from the superintendents who have evolved an exact system of nursing under difficulties as regards distance and mixed populations such as we have never had to confront. The chapter on "Reorganisation," showing the gain in time and efficiency of placing one nurse in each district, abolishing specialisation, and training all nurses in all kinds of work, is admirable and most timely. The sections which deal with the Nurses' Committee and the Supply Depôt are particularly good, and it is evident that American nursing is ahead of us in the matter of records and statistics, the labour of this business being largely taken out of the nurses' busy hands. This is a valuable book for the nurses' reference library.

**Prevention of Venereal Disease.** By Sir G. Archdall Reid, K.B.E., M.B., C.M., F.R.S.E., with an introductory chapter by Sir H. Bryan Donkin, M.D., F.R.C.P. (London: Wm. Heinemann (Medical Books), Ltd., Bedford Street. Price 15s. net.)

If the primary aim of this book is to impress upon the general public that really efficient measures must be taken, and taken promptly, to limit the spread of venereal infection, it should undoubtedly achieve its object, and we could recommend it on that score alone. But, at the same time, it should be known it not only expresses but also explains the views of the Society for the Prevention of Venereal Disease. An eminently strong case for their contentions is put forward, and whether the reader has already sided with one or other of the parties in the recent controversy, or whether he begins with an open mind on the subject, he will have gained a most useful insight to facts as they really are.

**Clinical Methods.** By Hutchison and Rainy. Seventh edition. (Cassell. Price 12s. 6d. net.)

A demand extending over twenty-seven years and calling for a seventh edition is evidence enough of a book's essential usefulness and virility. It only remains for the reviewer to state that in its latest appearance "Clinical Methods" retains all its old familiar features, whilst embracing the most up-to-date methods of investigation, particularly in respect of the heart and urine. The new plates, representing the blood in malaria and the affections of the fundus oculi and tympanic membrane, are admirable, and the book as a whole remains indispensable. To wish it success would be an impertinence, both in the old and modern senses of that word.

